# University and stakeholder partnerships to innovate in sport – the development of the South African Cricketers’ Association (SACA) career transition screening tool

**DOI:** 10.17159/2078-516X/2023/v35i1a15218

**Published:** 2023-06-05

**Authors:** S Hendricks, JP van Wyk, B Player, R Schlebusch

**Affiliations:** 1 Division of Physiological Sciences, Department of Human Biology, Faculty of Health Sciences, University of Cape Town, Cape Town, South Africa; 2 Health through Physical Activity, Lifestyle and Sport (HPALS) Research Centre, Department of Human Biology, Faculty of Health Sciences, University of Cape Town, Cape Town, South Africa; 3 Institute for Sport, Physical Activity and Leisure, Leeds Beckett University, Leeds, United Kingdom; 4 South African Cricketers’ Association, Cape Town, South Africa; 5 Sportsthink 360, Claremont, Cape Town, South Africa

**Keywords:** player welfare, athlete transitioning, cricket, innovation, stakeholder engagement, South Africa

## Abstract

In sports, the value and mutual benefit of university–stakeholder partnerships have been well-recognised. It has been argued that cricket has a unique set of challenges compared to other team sports. In 2016, the South African Cricketers’ Association (SACA) and the University of Cape Town established a partnership to (i) conduct novel research on professional cricketers and (ii) ensure SACA programmes and initiatives are informed by said research and/or the currently available literature. As the demand on professional cricketers has increased, so has the interest in their career transitioning. That is, how do professional cricketers manage stressors created by changes (or non-changes) throughout their playing careers? To help identify gaps for intervention as a cricketer transitions through their professional career, the purpose of this short report is to describe how a university–stakeholder partnership developed a career transitioning screening tool for professional cricketers in South Africa.

## The value of university–stakeholder partnerships

Innovation can be considered the successful implementation of a novel idea that creates value for some or all of its stakeholders. To be innovative and solve challenges that will benefit society, universities are strongly encouraged to engage and build partnerships with external constituencies, stakeholders and communities. In sport, the value and mutual benefit of university (researchers) –stakeholder partnerships have been well-recognised – for example, from the perspective of the university, stakeholder partnerships help to guide research questions and offer direct access to athletes and policymakers. From the stakeholder perspective, university partnerships may offer expertise and resources which otherwise may not have been accessible. Considering these benefits, frameworks to enhance university-stakeholder partnerships in sport have been proposed.^[1]^ In many cases, while a key focus of the university–stakeholder partnerships is research, several internal reports and tools are also produced. Typically though, only the research findings of a partnership are shared and published in the literature. In other words, the literature rarely describes the collaborative process between researchers and stakeholders in the development of tools that address the specific needs of the stakeholders.

## Cricket and its unique set of challenges

According to the International Cricket Council Global Market Research Report, cricket is one of the world’s most popular team sports, with 106 member countries, over 300 million male and female participants, and more than one billion fans. It has been argued that cricket has a unique set of challenges compared to other team sports. These include: how performance is appraised by primarily using individual game statistics (for example, runs scored), the different formats of the game (5-day tests, 50 overs (1-days) and 20 overs (T20), and extensive travel and time away from home. For example, an international cricket tour may last up to two months and require playing all formats of the game.^[2]^

## Career transitioning in sport

As the demand on players in professional cricket has increased over the years, so has the interest in their career transitioning. That is, how do professional cricketers manage stressors created by changes (or non-changes) throughout their playing career, and how do systems best support these players? Most of the work on career transitioning in sport, including cricket, focuses on players transitioning out of the sport (i.e. retiring athletes).^[3]^ More recently, coping within career transitioning (for example, progressing from youth to senior level, or semi-professional to professional level) has also been recognised as a key contributing factor to the long-term health and well-being of the player.^[3]^ At the same time, factors that contribute to a successful career transition (improved adaptation and management of transition and greater post-transition well-being) have also been identified.^[4]^ One way a player can improve the likelihood of a successful transition is to plan for the career transition and engage in it voluntarily.^[4]^

## Player associations and university partnerships

Player associations are organisations that represent the needs and interests of players within the respective sport and play a key role in the ecosystem of professional sport. One of the primary objectives of a player association is to promote and enhance the general health and well-being of its members (i.e. the players). In South Africa, the South African Cricketers’ Association (SACA) fulfils this objective for all professional cricketers. In 2016, SACA and the University of Cape Town (UCT) established a partnership to (i) conduct novel research on professional cricketers and (ii) ensure SACA programmes and initiatives are informed by said research and/or the currently available literature. From a university perspective, the partnership signifies a high degree of engaged scholarship for staff and students and the opportunity to produce high-impactful research. To conduct ongoing research, UCT approved a SACA Research Ethics Database in 2017. The first project of this university-stakeholder partnership was related to the mental health of professional cricketers.^[2, 5]^ Subsequent to this project, SACA sought to improve players’ career transitioning both within and out of professional cricket. Specifically, SACA required a screening tool that would help them to identify gaps for intervention as the cricketer transitions through their career. Using such a screening tool, SACA would work with the cricketer to help prepare and plan for any forthcoming career transition. The purpose of this short report is to describe how SACA and the UCT partnered to develop a career transitioning screening tool for professional cricketers in South Africa.

## Stakeholder criteria and context

South Africa has 17 professional cricket teams (Proteas men and women, eight Division 1 and seven Division 2) teams. SACA allocates each team a Player Development Manager (PDM), who supports the cricketer throughout their professional playing career and for a period after retiring. The objective of the PDM is to assist players with all non-cricket-related aspects; so players are well-rounded individuals and prepared for a life after a professional cricket career. For example, assisting cricketers to continue their studies while playing cricket. PDMs meet with all players regularly; however, given the scheduling demands of cricket, PDMs may be meeting under time constraints. Some players may not even be aware they are going through a within-career transition, rendering conversations about it difficult.

In 2019, considering the role of the PDM and the need to improve players’ career transitioning (both within and out of professional cricket), SACA proposed the development of a screening tool to raise career transitioning awareness among its players. More specifically, to assist PDMs in identifying whether a player is experiencing a career transition or not, and whether an intervention is required, i.e. linking the player to a service. During the early stages of development, it was agreed that the best form for the tool would be a screening questionnaire. A screening questionnaire would be easy to administer to teams and completing it would be quick and straightforward. PDMs would then use the player’s questionnaire responses to initiate the career transition conversation and link the player to the appropriate service. The main criterion during the development of the screening questionnaire though was that it had to be based on career transitioning literature. Two versions of the screening tool were developed – one for players experiencing (or about to experience) within career transitioning and one for players approaching retirement.

## Career transitioning screening tool development

Park et al. ^[6]^ conducted a systematic review of career transitioning in sport and identified 15 factors associated with career transition quality. Of these factors, we deduced three will be incorporated into the screening tool as it fell within SACA’s remit: these were career/personal development, educational status and financial status. Athletic identity was also the strongest factor associated with transition quality,^[6]^ and it was decided questions on athletic identity should be included in the screening tool. Keeping in mind the criteria to have a quick and straightforward tool to complete, the within-career – ‘Check-In’(Appendix 1) – career transition screening tool was focused on 13 questions based on previously published scales and questionnaires – the Athletic Identity Measurement Scale (5 questions: 1 on self-identity (Q1), 2 on exclusivity (Q2 and Q3), 1 on negative affectivity (Q4) and 1 on social identity (Q5),^[6]^ The Perceived Available Support in Sport Questionnaire (The PASS-Q) (1 question with four sub-sections, namely, Resources for Financial Advice and Planning, Resources for Emotional Support, Resources for Career Advice, Resources for Study Advice (Q9),^[7]^ and questions on transition preparedness (Q10–Q13). Questions 6–8 related to career transitioning awareness and asked cricketers if they have ever experienced a career transition (Q6), currently experiencing a career transition (Q7) or if they foresee a career transition (out of cricket) in the next 12 months (Q8). The tool for players approaching retirement – ‘Prepare for Landing’ (Appendix 2) – focused on 22 questions - 10 questions from the ‘Check-in’ tool, with an additional 12 questions based on the Athletes Retirement Decision Inventory(ARDI).^[8]^ Questions 11–15 were anti-pull factors, Q16–18 pull factors, Q19–Q20 anti-push factors, and Q21–Q22 were push factors. Both career transition screening tools also included a section for PDM notes and potential actions. After the screening tools were finalised, both questionnaires were reviewed by BP, a past player and current PDM, to ensure each question was clear and understandable. Both the ‘Check-In’ and ‘Prepare for Landing’ career transition screening tools are currently available to all SACA PDMs to use when required. At this stage, whether screening should be mandatory for all players is an ongoing discussion.

## Conclusion

The purpose of this short report was to describe how a university–stakeholder partnership developed screening tools that help to identify gaps for intervention as a cricketer transitions through their professional career. A key strength of the screening tool is that it was based on the current literature on career transitioning and uses established, validated questionnaires, while at the same time fulfilling the criteria of the stakeholder. That is the screening tools needed to be quick and straightforward to complete considering the context in which they would be implemented (time-constrained PDM-Player meetings). It is worth noting that the career transition screening tools were developed specifically for the needs and context of SACA. As such, while the university–stakeholder partnership approach described here can most certainly be used in other sports and stakeholder(s) settings, the career transition screening tools themselves may not be as generalisable, and therefore the screening tools may require adapting when implemented in other sports settings.

## Supplementary Information




