# Supplementary material for: University and stakeholder partnerships to innovate in sport – the development of the South African Cricketers’ Association (SACA) career transition screening tool
Source: S Afr J Sports Med. 2023 Jun 5;35(1):v35i1a15218. doi: 10.17159/2078-516X/2023/v35i1a15218 (PMC10798606; doi:10.17159/2078-516X/2023/v35i1a15218)
Supplement: Supplementary file 1 [file 2078-516X-35-v35i1a15218-s001.pdf]

SACA Career Transition Screening Tool (Check-in)

Player Name: **Team:** **Date:**

1. Being a cricketer is an important part of who I am.

1	2	3	4	5
**Strongly Disagree**	**Disagree**	**Neutral**	**Agree**	**Strongly Agree**

2. Cricket is the most important part of my life.

1	2	3	4	5
**Strongly Disagree**	**Disagree**	**Neutral**	**Agree**	**Strongly Agree**

3. I spend more time thinking about playing cricket than anything else.

1	2	3	4	5
**Strongly Disagree**	**Disagree**	**Neutral**	**Agree**	**Strongly Agree**

4. I could become depressed if I could not play cricket anymore.

1	2	3	4	5
**Strongly Disagree**	**Disagree**	**Neutral**	**Agree**	**Strongly Agree**

5. Other people see me as a cricketer.

1	2	3	4	5
**Strongly Disagree**	**Disagree**	**Neutral**	**Agree**	**Strongly Agree**

SACA
South African
Cricketers' Association

SACA Career Transition Screening Tool (Check-in)

6. Have you experienced a career transition during your cricket career?

Yes	No	Unsure

7. Do you feel you are currently experiencing a career transition?

Yes	No	Unsure

8. Do you foresee a career transition (out of cricket) in the next 12 months?

Yes	No	Unsure

9. Currently, to what extent do you feel you have the following : -

Resources for Financial Advice and Planning

1	2	3	4	5
**Not at all**	**Some extent**	**Moderate extent**	**Considerable extent**	**Great extent**

Resources for Emotional Support

1	2	3	4	5
**Not at all**	**Some extent**	**Moderate extent**	**Considerable extent**	**Great extent**

Resources for Career Advice

1	2	3	4	5
**Not at all**	**Some extent**	**Moderate extent**	**Considerable extent**	**Great extent**

SACA
South African
Cricketers' Association

SACA Career Transition Screening Tool (Check-in)

Resources for Study Advice

1	2	3	4	5
**Not at all**	**Some extent**	**Moderate extent**	**Considerable extent**	**Great extent**

10. If my cricket career ended today, I will be emotionally prepared to handle the career transition.

1	2	3	4	5
**Strongly Disagree**	**Disagree**	**Neutral**	**Agree**	**Strongly Agree**

11. If my cricket career ended today, I will be financially prepared to handle the career transition.

1	2	3	4	5
**Strongly Disagree**	**Disagree**	**Neutral**	**Agree**	**Strongly Agree**

12. If my cricket career ended today, I will have the necessary motivation and skills to find meaningful employment.

1	2	3	4	5
**Strongly Disagree**	**Disagree**	**Neutral**	**Agree**	**Strongly Agree**

13. If my cricket career ended today, I will be financially stable for the next 6 months.

1	2	3	4	5
**Strongly Disagree**	**Disagree**	**Neutral**	**Agree**	**Strongly Agree**

SACA Career Transition Screening Tool (Check-in)

PDM Notes:

Potential Actions:

SACA
South African
Cricketers' Association
