# Supplementary material for: University and stakeholder partnerships to innovate in sport – the development of the South African Cricketers’ Association (SACA) career transition screening tool
Source: S Afr J Sports Med. 2023 Jun 5;35(1):v35i1a15218. doi: 10.17159/2078-516X/2023/v35i1a15218 (PMC10798606; doi:10.17159/2078-516X/2023/v35i1a15218)
Supplement: Supplementary file 2 [file 2078-516X-35-v35i1a15218-s002.pdf]

SACA Career Transition Screening Tool (Preparation for Landing)

Player Name: **Team:** **Date:**

1. Being a cricketer is an important part of who I am.

1	2	3	4	5
**Strongly Disagree**	**Disagree**	**Neutral**	**Agree**	**Strongly Agree**

2. Cricket is the most important part of my life.

1	2	3	4	5
**Strongly Disagree**	**Disagree**	**Neutral**	**Agree**	**Strongly Agree**

3. I spend more time thinking about playing cricket than anything else.

1	2	3	4	5
**Strongly Disagree**	**Disagree**	**Neutral**	**Agree**	**Strongly Agree**

4. I could become depressed if I could not play cricket anymore.

1	2	3	4	5
**Strongly Disagree**	**Disagree**	**Neutral**	**Agree**	**Strongly Agree**

5. Other people see me as a cricketer.

1	2	3	4	5
**Strongly Disagree**	**Disagree**	**Neutral**	**Agree**	**Strongly Agree**

SACA
South African
Cricketers' Association

SACA Career Transition Screening Tool (Preparation for Landing)

6. Currently, to what extent do you feel you have the following: -

Resources for financial advice and planning

1	2	3	4	5
**Not at all**	**Some extent**	**Moderate extent**	**Considerable extent**	**Great extent**

Resources for Emotional Support

1	2	3	4	5
**Not at all**	**Some extent**	**Moderate extent**	**Considerable extent**	**Great extent**

Resources for Career Advice

1	2	3	4	5
**Not at all**	**Some extent**	**Moderate extent**	**Considerable extent**	**Great extent**

Resources for Study Advice

1	2	3	4	5
**Not at all**	**Some extent**	**Moderate extent**	**Considerable extent**	**Great extent**

7. If my cricket career ended today, I will be emotionally prepared to handle the career transition.

1	2	3	4	5
**Strongly Disagree**	**Disagree**	**Neutral**	**Agree**	**Strongly Agree**

SACA
South African
Cricketers' Association

SACA Career Transition Screening Tool (Preparation for Landing)

8. If my cricket career ended today, I will be financially prepared to handle the career transition.

1	2	3	4	5
**Strongly Disagree**	**Disagree**	**Neutral**	**Agree**	**Strongly Agree**

9. If my cricket career ended today, I will have the necessary motivation and skills to find meaningful employment.

1	2	3	4	5
**Strongly Disagree**	**Disagree**	**Neutral**	**Agree**	**Strongly Agree**

10. If my cricket career ended today, I will be financially stable for the next 6 months.

1	2	3	4	5
**Strongly Disagree**	**Disagree**	**Neutral**	**Agree**	**Strongly Agree**

11. I am afraid of changes that will be generated from transitioning out of playing professional cricket.

1	2	3	4	5
**Strongly Disagree**	**Disagree**	**Neutral**	**Agree**	**Strongly Agree**

12. I am afraid of not being able to adapt to another job.

1	2	3	4	5
**Strongly Disagree**	**Disagree**	**Neutral**	**Agree**	**Strongly Agree**

SACA
South African Cricketers' Association

SACA Career Transition Screening Tool (Preparation for Landing)

13. I am concerned about my mental health after retiring from professional cricket.

1	2	3	4	5
**Strongly Disagree**	**Disagree**	**Neutral**	**Agree**	**Strongly Agree**

14. I am concerned about my physical health after retiring from professional cricket.

1	2	3	4	5
**Strongly Disagree**	**Disagree**	**Neutral**	**Agree**	**Strongly Agree**

15. I am afraid of no longer having contact with the world of professional cricket after retiring.

1	2	3	4	5
**Strongly Disagree**	**Disagree**	**Neutral**	**Agree**	**Strongly Agree**

16. I am looking forward to setting new professional career goals after retirement.

1	2	3	4	5
**Strongly Disagree**	**Disagree**	**Neutral**	**Agree**	**Strongly Agree**

17. I am looking forward to working on new projects (outside of playing cricket).

1	2	3	4	5
**Strongly Disagree**	**Disagree**	**Neutral**	**Agree**	**Strongly Agree**

SACA
South African Cricketers' Association

SACA Career Transition Screening Tool (Preparation for Landing)

18. I am looking forward to spending time on other interests (outside of cricket).

1	2	3	4	5
**Strongly Disagree**	**Disagree**	**Neutral**	**Agree**	**Strongly Agree**

19. I still enjoy playing professional cricket.

1	2	3	4	5
**Strongly Disagree**	**Disagree**	**Neutral**	**Agree**	**Strongly Agree**

20. I feel I am too young to retire from professional cricket.

1	2	3	4	5
**Strongly Disagree**	**Disagree**	**Neutral**	**Agree**	**Strongly Agree**

21. I am dissatisfied with the current cricket structures.

1	2	3	4	5
**Strongly Disagree**	**Disagree**	**Neutral**	**Agree**	**Strongly Agree**

22. I received assistance in preparing for the end of my cricket career.

1	2	3	4	5
**Strongly Disagree**	**Disagree**	**Neutral**	**Agree**	**Strongly Agree**

SACA
South African
Cricketers' Association

SACA Career Transition Screening Tool (Preparation for Landing)

PDM Notes:

Potential Actions:

SACA
South African
Cricketers' Association
